# Controlling crystallites orientation and facet exposure for enhanced electrochemical properties of polycrystalline MoO_3_ films

**DOI:** 10.1038/s41598-023-43800-9

**Published:** 2023-10-04

**Authors:** Konrad Trzciński, Zuzanna Zarach, Mariusz Szkoda, Andrzej P. Nowak, Katarzyna Berent, Mirosław Sawczak

**Affiliations:** 1 Faculty of Chemistry, Gdańsk University of Technology, Narutowicza 11/12, 80-233 Gdańsk, Poland; 2 Advanced Materials Center, Gdańsk University of Technology, Narutowicza 11/12, 80-233 Gdańsk, Poland; 3 Centre for Plasma and Laser Engineering, The Szewalski Institute of Fluid Flow Machinery, Fiszera 14, 80-231 Gdańsk, Poland; 4 Academic Centre for Materials and Nanotechnology, AGH University of Krakow, Mickiewicza 30 Ave, 30-059 Kraków, Poland

**Keywords:** Materials for energy and catalysis, Design, synthesis and processing, Surfaces, interfaces and thin films

## Abstract

This study focuses on the development and optimization of MoO_3_ films on commercially available FTO substrates using the pulsed laser deposition (PLD) technique. By carefully selecting deposition conditions and implementing post-treatment procedures, precise control over crystallite orientation relative to the substrate is achieved. Deposition at 450 °C in O_2_ atmosphere results in random crystallite arrangement, while introducing argon instead of oxygen to the PLD chamber during the initial stage of sputtering exposes the (102) and (011) facets. On the other hand, room temperature deposition leads to the formation of amorphous film, but after appropriate post-annealing treatment, the (00k) facets were exposed. The deposited films are studied using SEM and XRD techniques. Moreover, electrochemical properties of FTO/MoO_3_ electrodes immersed in 1 M AlCl_3_ aqueous solution are evaluated using cyclic voltammetry and electrochemical impedance spectroscopy. The results demonstrate that different electrochemical processes are promoted based on the orientation of crystallites. When the (102) and (011) facets are exposed, the Al^3+^ ions intercalation induced by polarization is facilitated, while the (00k) planes exposure leads to the diminished hydrogen evolution reaction overpotential.

## Introduction

**Figure 1:**
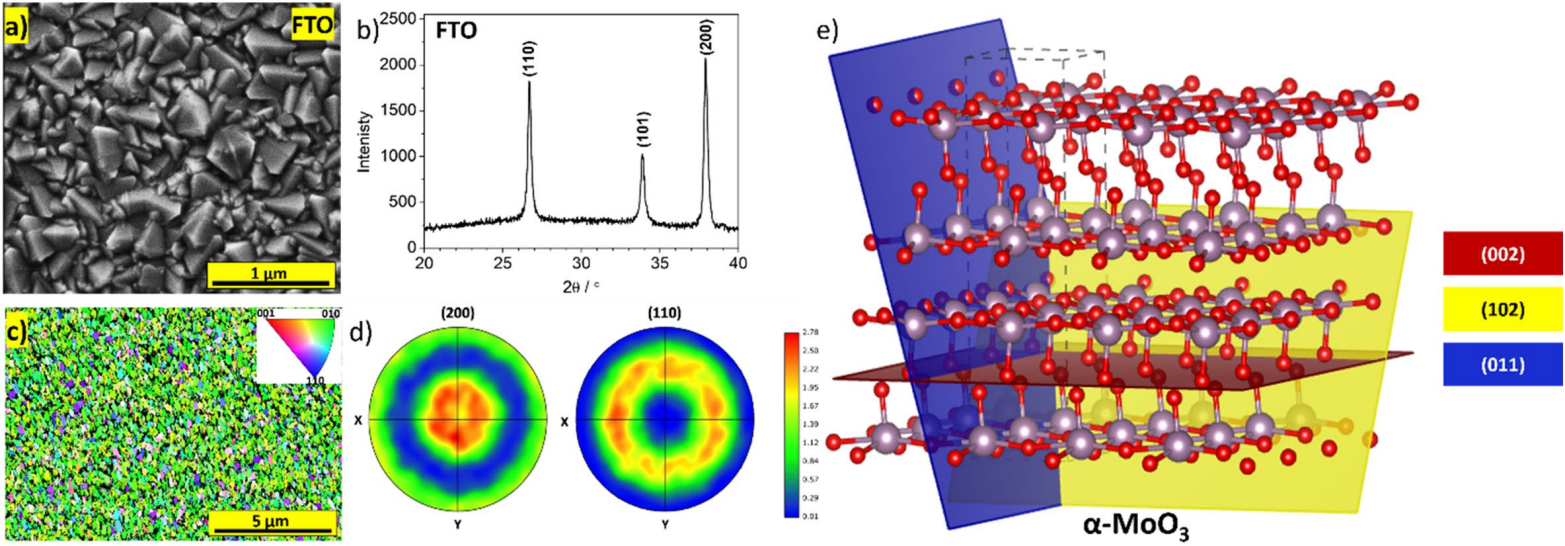
(**a**) SEM micrograph and (**b**) XRD pattern of the FTO substrate; (**c**) Inverse pole figure and (**d**) pole figures for (200) and (110) planes of the FTO substrate; (**e**) α-MoO_3_ crystal structure with (002), (102), and (011) planes highlighted.

The orientation of crystallites relative to the substrate, as well as the exposed crystal facets, affect the properties of deposited films. The phenomenon is universal and has been reported for various materials, including BiVO_4_^1^, WO_3_^2^, TiO_2_^3^, ZnO^4^, Bi_2_O_3_^5^, and others. The ability to control the exposure of specific crystal facets can be beneficial for applications in photoelectrochemistry^3^, energy storage and conversion^6^, photocatalysis^7^, and sensing^8^. In general, the approach of controlling the crystals' orientation may be advantageous wherever the crystalline material exhibits anisotropic properties or possess active centers on specific facets. In the case of film deposition, the most common way to affect the crystal orientation of sputtered materials is by selecting an appropriate substrate that promotes growth in the desired direction due to lattice matching. This method enables the production of high-quality films, however, such procedures limit the possible applications of the deposited layers due to the properties of those single-crystalline substrates, such as non-conductivity, opaqueness, high price, or instability in operating conditions. Thus, it would be advantageous to develop methods for affecting the crystal orientation on randomly oriented substrates, such as FTO (fluorine-doped tin oxide). An exemplary SEM image and XRD pattern of an FTO substrate that confirms the random arrangement of crystallites is shown in Fig. 1a,b. The crystal orientations of the FTO were investigated by electron backscatter diffraction (EBSD). Figure 1c,d presents the inverse pole figure (IPF) and pole figures for (200) and (110) planes. The FTO substrate shows broad spots for (200) and ring-shaped patterns for (110) planes, respectively, which is consistent with the results obtained from XRD. FTO glass possesses desirable characteristics such as transparency, conductivity, thermal and chemical stability over a wide range of pH^9^, making it suitable for a wide range of applications. Previous reports have demonstrated that BiVO_4_, when deposited on single crystal YSZ (yttria-stabilized zirconia) substrate due to lattice matching, exhibits (004) facet exposition, which is highly convenient for photoelectrochemical water splitting^10^. However, a similar effect has also been achieved using high-temperature pulsed laser deposition, even on an FTO polycrystalline substrate^11^.

The primary objective of this paper is to investigate and customize the properties of MoO_3_ films deposited on commercially available FTO substrates. Through the exploration of various deposition techniques and post-treatment methods, the aim is to optimize and tailor the characteristics of the MoO_3_ films for specific applications. α-MoO_3_ is a metal oxide characterized by a layered structure and is distinguished by the presence of van der Waals (vdW) gaps^12^. These vdW gaps contribute to the unique physical and chemical properties exhibited by α-MoO_3_. Understanding the relationship between crystal structure and anisotropy is crucial for optimizing the functionality and performance of α-MoO_3_ in various fields, including energy storage, sensing, and catalysis. Figure 1e depicts the representation of the crystal unit with highlighted facets, illustrating that the van der Waals gaps are parallel to the (00k) planes. This crystal structure suggests that the material can exhibit anisotropy in properties such as ion conductivity, and demonstrate varying activity on specific crystal facets. And indeed, previous studies have proven that α-MoO_3_ exhibits anisotropy, particularly in catalytic properties^13^. Moreover, this material is also commonly investigated as an electrode material for batteries and electrochemical capacitors^14,15^, but also for photocatalysts^16^, electrochromic films^17^, and gas sensing materials^18^. Hence, it is reasonable to develop methods of MoO_3_ deposition that enable the control of crystals orientation and exposure of specific crystal facets on the same type of polycrystalline substrate. Until recently, various methods of MoO_3_ deposition have been proposed, including PLD^19^, spray pyrolysis^20^, RF sputtering^21^, and electrodeposition^22^. The orientation of the deposited crystals is found to depend on the choice of substrates and deposition parameters, as indicated by the literature. Pulsed laser deposition of MoO_3_ on Si (111) wafers results in the formation of α-MoO_3_ with exposed (00k) planes. On alumina polycrystalline substrates, the same deposition technique leads to the formation of suboxides, but subsequent post-annealing significantly enhances the intensities of (102) and (011) reflections^23^. On the other hand, spray-pyrolysis of molybdenum trioxide on glass substrates, conducted at an appropriate temperature using MoCl_5_ and (NH_4_)_6_Mo_7_O_24_·4H_2_O solutions as precursors, allows the exposure of (00k) planes^20,24^, whereas RF sputtering of MoO_3_ on glass and silicon substrates results in amorphous film, but a post-annealed film consists of both, α- and β-MoO_3_ phases^21^. In this study, we demonstrate the deposition of MoO_3_ polycrystalline films with desired orientation on FTO substrates using the PLD system.

## Experimental part

The FTO substrates (7 Ω/sq) were purchased from Merck. The cleaning procedure involved ultrasonic treatment of the substrates in acetone, ethanol, and isopropanol (all from POCH) for approximately 20 min each. Subsequently, the substrates were dried using a stream of air. Prior to deposition, the FTO pieces underwent treatment with oxygen plasma using plasma cleaner (Femto, Diener). The deposition process was conducted in a developed PLD chamber, where the laser beam was directed perpendicularly onto the metallic molybdenum target, while the substrates were positioned parallel to the beam at a distance of 10 mm from the target. A 266nm, 6ns pulse-duration Nd:YAG laser (Brilliant B, Quantel) with an FHG module was employed for the ablation process. The laser’s energy density was set at approximately 2.5 J cm^2^. Further technical details about the deposition system can be found in our previous paper^25^. Samples deposited at room temperature were subsequently annealed in oven (FCF 1SP, Czylok) under an air atmosphere. The metallic Mo thin film was deposited using magnetron sputtering system (Q150T, Quorum). The morphology of the deposited films was examined using scanning electron microscope (SU3500, Hitachi) at a 10 kV accelerating voltage. Energy dispersive x-ray spectroscopy (EDX) measurements were performed using an EDAX Genesis APEX 2i with an ApolloX SDD spectrometer. XRD patterns were collected using a Rigaku Intelligent X-ray diffraction system Smartlab with Cu Kα radiation. The patterns were recorded in a range of 10–40°. EBSD analysis was performed using scanning electron microscope (Versa 3D, FEI), operating at an accelerating voltage of 15 kV, equipped with CMOS detector (Symmetry S2, Oxford Instruments). The collected EBSD data was post-processed using the AZtecCrystal software. All electrochemical experiments were performed using a potentiostat (Vertex, Ivium) in a three-electrode cell, where FTO/MoO_3_, Ag/AgCl (3 M KCl), and platinum mesh served as working electrode, reference electrode, and counter electrode, respectively. The measurements were performed in 1 M AlCl_3_ (AlCl_3_·6H_2_O, 99%, Alfa Aesar) aqueous solution. The pH of the prepared electrolyte was 2.2. The geometric surface area of the electrode in contact with the electrolyte was approximately ~ 0.2 cm^2^.

## Results and discussion

### Deposition of MoO_3_ with (011) and (102) planes exposed

**Figure 2:**
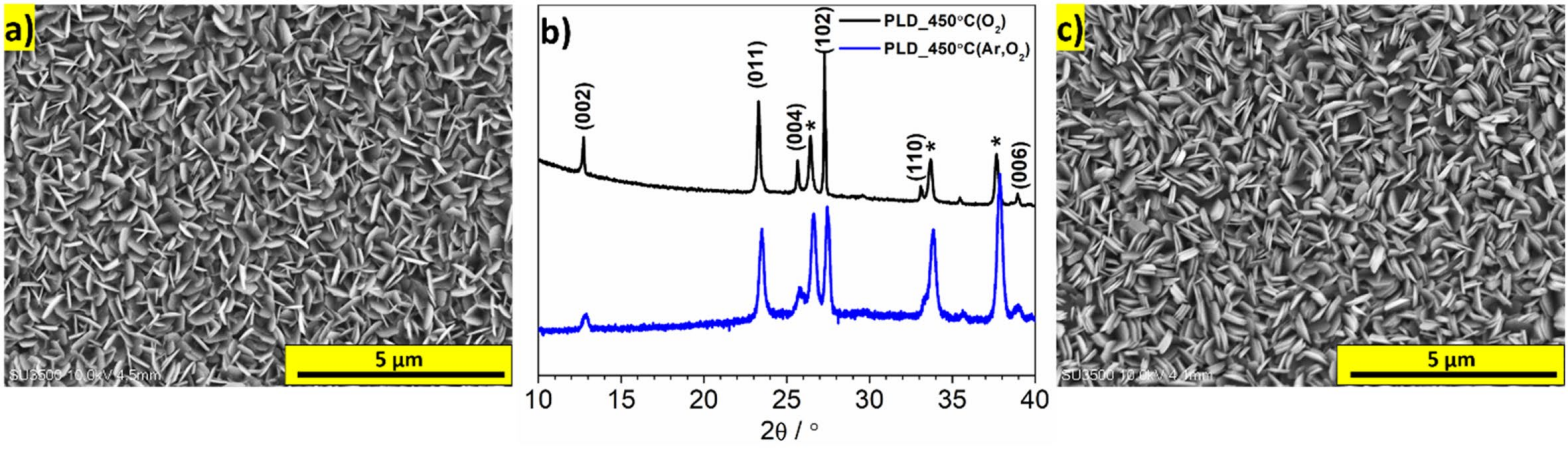
(**a**) SEM micrograph of the PLD_450 °C(O_2_) film; (**b**) XRD patterns of the PLD_450 °C(O_2_) and PLD_450 °C(Ar,O_2_) materials (reflections labeled with “*” origin from FTO); (**c**) SEM micrograph of the PLD_450 °C(Ar,O_2_) film.

First, the MoO_3_ material was deposited at 450 °C in O_2_ atmosphere (0.5 mbar) for 120 min. The resulting MoO_3_ film uniformly covered the entire surface of the FTO substrate. The SEM micrograph of the deposited film is presented in Fig. 2a. The polycrystalline film consisted of thin plates with sizes up to 500 nm. The crystallites appeared to be predominantly oriented perpendicular to the substrate, although a mixture of different orientations was observed. The XRD pattern showed that the most intense reflexes originated from the (102) and (011) planes, while the (002), (004), and (006) planes were also clearly visible (see Fig. 2b, PLD_450 °C(O_2_)). Additionally, deposition was performed at different temperatures, and deposition at 400 °C also resulted in a random orientation of crystallites, while deposition at 500 °C did not lead to the formation of a distinct layer (see Fig. S1a and b, respectively). In order to achieve a more ordered arrangement of α-MoO_3_ crystallites, slight modifications were made to the deposition parameters. The deposition process commenced at room temperature in an argon atmosphere. After 10 min, oxygen was introduced into the chamber, and the temperature was raised to 450 °C (sample labeled as PLD_450 °C(Ar,O_2_)). The SEM image of resulting layer is presented in Fig. 2c. The morphology of the layer was relatively similar, consisting of crystallites with a similar appearance. However, upon closer examination, it was observed that the majority of crystallites were perpendicular to the FTO substrate. Moreover, the intensities of the reflections from the (00k) planes were significantly diminished, and the peaks appear broader in comparison with the conventionally deposited film pattern (Fig. 2b). Based on this results, it can be concluded that the exposed facets observed in the SEM image were (011) and (102), while the (00k) planes were oriented perpendicular to the substrate. Considering this findings, it can be inferred that vdW gaps are also perpendicular to FTO and the direction of crystallites growth is related to the initial stage of the process when metallic Mo is deposited in argon atmosphere. To validate this hypothesis, a 20 nm layer of metallic Mo was deposited on FTO using a magnetron sputtering machine, and then the FTO/Mo was used as a substrate for MoO_3_ deposition using PLD (O_2_ atmosphere, 120 min, 450 °C). The resulting layer exhibited a similar morphology and exposed (102) and (011) facets (SEM micrograph is shown in Fig. S1c). Thus, the presence of a thin Mo film during the initial stage of MoO_3_ deposition is crucial for obtaining a film with exposed (011) and (102) facets.

In order to enhance the readability of the manuscript, the sample deposited at a temperature of 450 °C in a two-step process (in Ar and O_2_) is labeled as MoO_3__(011)&(102).

### Deposition of MoO_3_ with (00k) planes exposed

**Figure 3:**
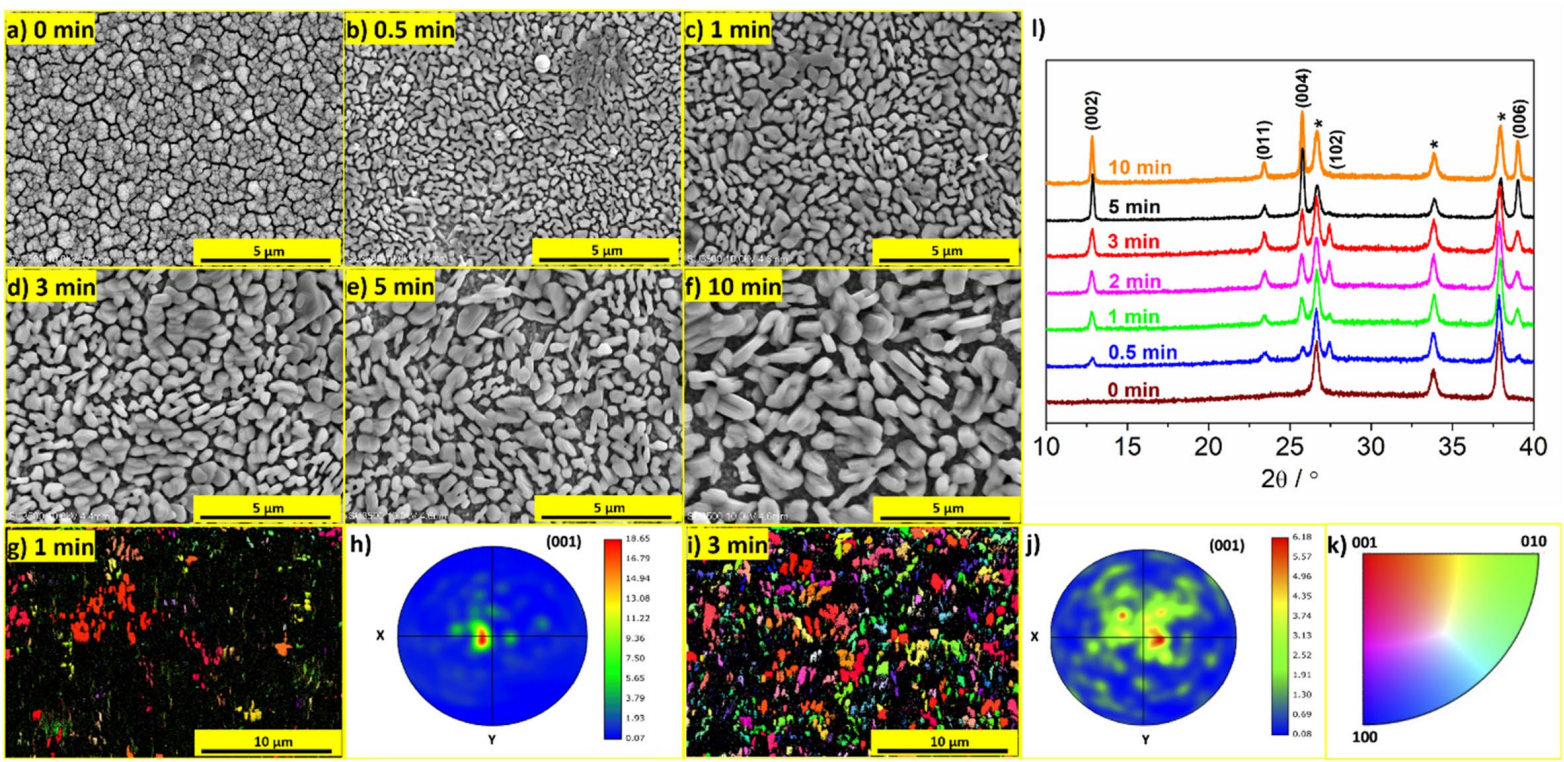
(**a**–**f**) SEM micrographs of MoO_3_ films deposited on the FTO substrate at room temperature for 120 min, followed by “rapid” post-annealing for various durations; EBSD inverse pole figure (IPF) map and pole figures for annealed MoO_3_ film at (**g**, **h**) 1 min, and (**i**, **j**) 3 min; (**k**) The color code representing the crystal orientation; (**l**) The XRD patterns of MoO_3_ films.

The same PLD system, substrates, and target were used to obtain a MoO_3_ film with differently oriented crystallites and exposed (00k) planes. The deposition process was performed at room temperature for the same duration (120 min) and under the same oxygen pressure (0.5 mbar). The SEM image of the as-deposited film is presented in Fig. 3a, where no distinguishable crystallites can be observed, indicating an amorphous nature of as-deposited film. To crystallize the layer, the sample was annealed in an air atmosphere. However, conventional annealing at 450 °C for 4 h with a slow heating rate resulted in the formation of relatively large crystallites that poorly covered the substrate and exhibited completely random arrangement, see Fig. S2a. Nevertheless, through optimization of the crystallization procedure, it was possible to obtain a film with exposed (00k) facets. The amorphous films were directly placed in a heated oven and annealed at 575 °C for varying periods of time (0–20 min). The selected temperature was limited by thermal stability of the FTO substrates (lower temperatures did not yield the expected effect). The SEM images of the post-annealed films are presented in Fig. 3b–f. The morphology of the films underwent significant changes with increasing annealing time. After 0.5,1, and 3 min of heating, areas can be found where the grains merge together. EBSD measurements were performed for samples annealed at different times. Figure 3g–k shows the IPF maps, corresponding pole figures, and color code representing crystal orientation of MoO_3_ films annealed at 1 min and 3 min. The analyses of the pole figures obtained from EBSD data confirm a preferential orientation of the grains with the [001] direction. It is clearly visible that in the first stage of annealing, the crystallites start to grow parallel to the (00k) planes. The black areas in the IPF maps are unindexed areas resulting from the presence of amorphous regions, the content of which is reduced during prolonged annealing. The appearance of the crystallites growing in other directions is observed after 3 min of annealing, however, most of the crystallites choose a preferential direction growth in such a way that (00k) planes are exposed. Prolonging the annealing time resulted in fully polycrystalline layers, with larger crystallites formed as the heating duration increased. Notably, after 20 min, the crystal size expanded to several micrometers, causing partial exposure of the FTO substrate surface, as it is shown in Fig. S2b. In the studied case, where the aim is to obtain a film of deposited material, the observed effect of patchy layer formation is unfavorable. Extending the annealing time to 60 min at a temperature of 575 °C revealed an intriguing phenomenon concerning the behavior of MoO_3_. It appeared that the material underwent sublimation from the FTO substrate, as evidenced by the absence of MoO_3_ on part of the FTO surface (Fig. S3a). This sublimation phenomenon was unexpected since it is generally claimed that the sublimation temperature of MoO_3_ is higher than 780 °C^26^. However, it has been reported that in the case of the MoO_3_ nanoplates, the sublimation occurred even at prolonged heating at temperatures higher than 400 °C^27^. This sublimation phenomenon highlights the sensitivity of MoO_3_ in a form of thin films to elevated temperatures and emphasizes the importance of carefully controlling the annealing conditions to achieve the desired layer deposition and maintain film integrity. Furthermore, in the case presented here, the material deposited at room temperature appears to be amorphous. The proposed crystallization method could result in the sublimation of any remaining uncrystallized material. The energetically unfavorable nature of amorphous materials, compared to ordered crystals, may reduce the energy requirements of thermally activated processes, such as sublimation. Based on these findings, a post-annealing duration of 5 min was determined to be optimal for the sample, labeled as MoO_3__(00k). A digital photo of the sample is shown in Fig. S3b for comparison with the sample annealed for 60 min. The morphology of the films presented in Fig. 3 differs significantly from the morphology of MoO_3_ deposited at 450 °C. In contrast to the crystallites deposited at high temperatures, the crystallites in post-annealed samples after room temperature deposition appeared to be oriented parallel to the FTO substrate. The XRD patterns of the films after annealing at 575 °C are shown in Fig. 3g. As expected, the as-deposited layer was amorphous, with only reflexes originating from the FTO substrate. After 30 s of heating, low-intensity reflections characteristic of α-MoO_3_ appeared and as the heating time increased, the intensity of these signals grew, indicating a more crystalline material. The XRD results are in agreement with the EBSD analysis. Moreover, after 5 min of annealing, the intensity of the MoO_3_ peaks exceeded that of the peaks originating from the FTO substrate. Notably, the intensities of the peaks did not increase uniformly as the reflexes originating from the (002), (004), and (006) planes exhibited much higher intensity compared to the (011) plane, while the peak from (102) plane nearly disappeared. On the basis of XRD results, it can be inferred that the majority of the crystallites is arranged in such a way that the (00k) planes are exposed. Considering the crystal structure of α-MoO_3_ (see Fig. 1a), the vdW gaps for this type of samples were parallel to the substrate. The post-annealing of amorphous MoO_3_ was also performed at lower temperatures (400 and 450 °C) using the same method of directly placing the samples in the hot oven for 60 min. The obtained films were polycrystalline and uniformly covered the substrate (no sublimation of MoO_3_ was observed), see Fig. S4a and b. However, the XRD results demonstrated that exposure of the (00k) plane was not achieved at this temperature (Fig. S4c). Therefore, to obtain a MoO_3_ film with the (00k) planes exposed, the “rapid” annealing at 575 °C for a short period of time was required.

Two types of the samples, labeled as MoO_3__(011)&(102) and MoO_3__(00k) were selected for further comparison of their properties using electrochemical methods.

### Electrochemical properties

**Figure 4:**
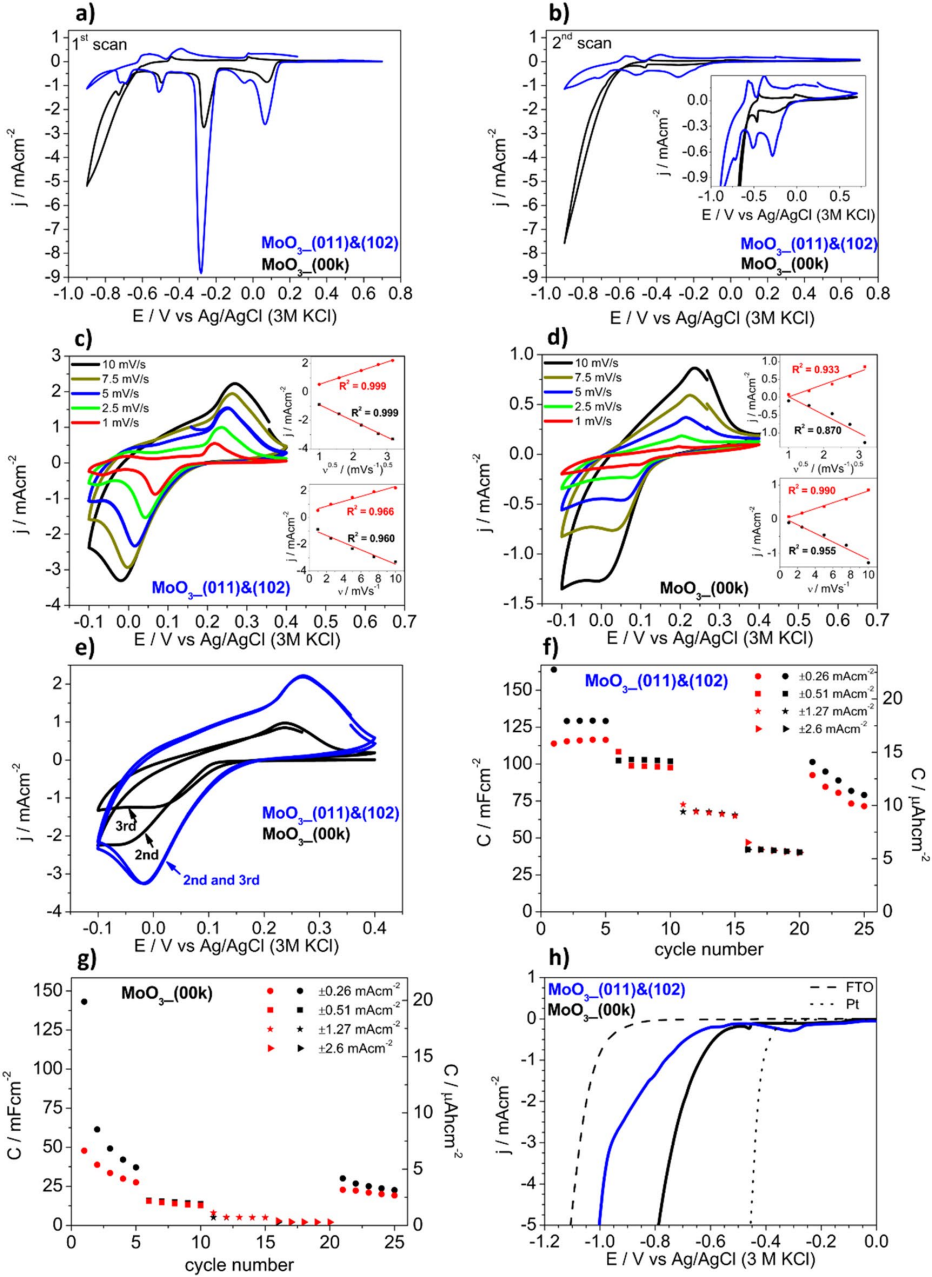
(**a**) The 1st and (**b**) the 2nd cv scans (*v* = 2.5 mVs^−1^) of MoO_3__(001)&(102) and MoO_3__(00k); (**c**) and (**d**) Comparison of cv 2nd scans of MoO_3__(001)&(102) and MoO_3__(00k) recorded at different scan rates (insets: *j* = *f*(*ν*) and *j* = *f*(*ν*^0.5^)); (**e**) Comparison of 2nd and 3rd cv scans (*v* = 10 mVs^-1^); (**f**) and (**g**) Comparison of the capacitance determined from galvanostatic charge/discharge cycles for MoO_3__(001)&(102) and MoO_3__(00k) (from − 0.1 to 0.4 V vs. Ag/AgCl (3 M KCl), black and red symbols stands for cathodic and anodic scans, respectively); (**h**) lv curves recorded at potential range suitable for HER for MoO_3__(001)&(102), MoO_3__(00k), bare FTO, and Pt (*v* = 2.5 mVs^−1^). All measurements were performed in 1 M AlCl_3_.

Both types of samples were subjected to investigation using electrochemical methods in a 1 M AlCl_3_ aqueous electrolyte. Previous studies have already reported the satisfactory electrochemical properties of MoO_3_ in such an electrolyte^28^. First, cyclic voltammetry curves were recorded to compare the behavior of the MoO_3__(011)&(102) and MoO_3__(00k) electrode materials, as shown in Fig. 4a,b. Generally, during the first scan, both samples exhibited cathodic peaks occurring at the same potentials. Notably, the current density for the MoO_3__(011)&(102) sample was higher than that of the MoO_3__(00k) one. These cathodic peaks correspond to the reduction of Mo(VI) centers and simultaneous cation insertion. The complexity of the AlCl_3_ aqueous solution chemistry, including hydrolysis, makes it unclear which form of ion is being intercalated. However, the ex-situ EDX measurements confirms the presence of Al in the sample after cathodic polarization (− 0.1 V vs. Ag/AgCl (3 M KCl)), as it is shown in Fig. S5, indicating that the observed electroactivity is related to the insertion of Al-containing cations into the MoO_3_ structure. The presence of chlorides was also detected, suggesting that the electrolyte was simply adsorbed on the electrode surface, however, the excess of Al (considering the stoichiometry of AlCl_3_) clearly indicates the incorporation of Al-containing ions into the structure. Regarding the shape of the cyclic voltammetry curves, both samples exhibited clear irreversibility of the electrochemical processes. MoO_3_ is sensitive to the potential range during polarization^29^, indicating that cations are likely irreversibly intercalated into the electrode material structure. The cathodic peak observed during the 1st scan at around E = 0.05 V versus Ag/AgCl (3 M KCl) disappeared and was not seen during the 2nd scan. Similar behavior has already been reported for MoO_3_-based electrodes tested in Mg^2+^-containing electrolytes^30^. In the case of the sample with (011) and (102) facets exposed, the electrochemical activity related to cation intercalation/deintercalation was clearly observed during 2nd scan, as well. The peaks registered for the electrode material with (00k) planes exposed almost disappeared, but a similar shape of cyclic voltammogram has been reported before^31^. This suggests that the MoO_3__(011)&(102) sample, with vdW gaps perpendicular to the substrate, is a more suitable electrode material for energy storage due to the occurrence of intercalation/deintercalation processes during subsequent scans. In such wide range of potentials, the electrodes exhibited very poor stability during cycling, thus measurements were also performed in a narrower range to avoid irreversible intercalation. The cyclic voltammetry curves measured from − 0.1 to 0.4 V (vs. Ag/AgCl (3 M KCl) at different scan rates are shown in Fig. 4c,d. For the MoO_3__(011)&(102) sample, the well-shaped peaks related to cations intercalation/deintercalation were recorded for all scan rates. The perfect linear fit of the current density peaks versus the square root of the scan rate confirms that the kinetics of the process is diffusion controlled, see the inset of Fig. 4c. In the case of the sample with the (00k) planes exposed, the peaks (especially cathodic ones) are broadened and do not have a clear maximum, suggesting poorer kinetics in comparison with previous one, as it is shown in Fig. 4d. Additionally, both *j* = *f*(*ν*) and *j* = *f*(*ν*^0.5^) are not linear, see the inset of Fig. 4d. The MoO_3__00k, in contrast to MoO_3__(011)&(102), shows a decrease in current densities during subsequent polarization cycles, see the comparison of the 2nd and 3rd cv scans in Fig. 4e. Thus, not linear behavior is probably related to the electrode material deactivation that progresses during the polarization cycles, not with the nature of the electrochemical process itself. The potential usability of electrodes in energy storage devices was also tested during galvanostatic charge/discharge measurements. The measurements were performed from − 0.1 to 0.4 V (vs Ag/AgCl (3 M KCl)). The obtained results are presented in Fig. 4f,g. The capacitance (and capacity) estimated from the first, cathodic curve is in the same range for both types of electrodes. However, at the lowest current density, the capacitance of MoO_3__(011)&(102) stabilizes at values 4 times higher in comparison with MoO_3__(00k). At higher current densities, the capacitance remains at a reasonable level only for the sample with crystal orientation for which an intercalation/deintercalation is facilitated ((001) and (102) exposed). Such behavior may be simply related to the poor stability of the MoO_3__(00k) during cycling. However, the very low capacitance measured at higher current densities can be also affected by sluggish kinetics of the ion intercalation, while vdW gaps are parallel to the substrate.

On the other hand, as shown in the polarization curves, the MoO_3__(00k) sample exhibited a significantly lower overpotential for the hydrogen evolution reaction (HER) as presented in Fig. 4h. The results for bare Pt disc and FTO substrate are shown for comparison. The results prove that the MoO_3_ sample with exposed (00k) planes was characterized by higher electrocatalytic activity and faster kinetics in promoting the HER. Enhanced electrocatalytic properties can be attributed to a hindered proton intercalation process. The limited insertion of cations into the MoO_3_ structure may consequently result in a diminished overpotential for H^+^ reduction when compared to an electrode, where intercalation is facilitated. The electrocatalytic properties of MoO_3_ towards HER have already been reported^32^. Moreover, it has been presented that MoO_3_ in the form of nanobelts, with (00k) planes exposed, exhibits superior properties for H_2_ evolution compared to commercially available MoO_3_^33^.

**Figure 5:**
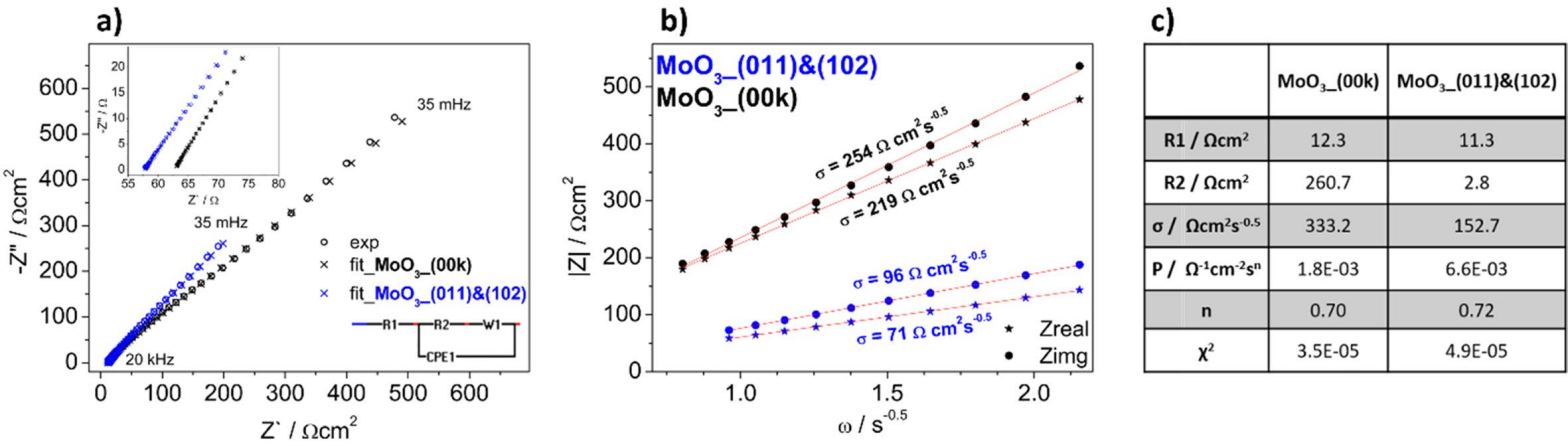
(**a**) Nyquist plots and (**b**) Z = f(ω^−0.5^) plots for the MoO_3__(011)&(102) and MoO_3__(00k) electrode materials, recorded at E = − 0.1 V versus Ag/AgCl (3 M KCl); (**c**) The results of EIS fitting for both electrodes.

Additionally, electrochemical impedance spectroscopy measurements were performed and the comparison of spectra recorded during cathodic polarization (E = − 0.1 V vs. Ag/AgCl (3 M KCl)) is shown in Fig. 5a. The measurements were performed at the potential that Al-containing ions are intercalated to the electrode material. As expected, due to the mechanism of charge storage in MoO_3_^34^, there is a range of frequencies where the spectra exhibit diffusional behavior. Thus, the Warburg coefficients were estimated as the slope of Z = *f*(ω^−0.5^) function, as shown in Fig. 5b. This analysis suggests that apparent diffusion coefficient of cations (it is assumed that it is Al^3+^ diffusion) is higher for the MoO_3__(011)&(102) electrode material compared to the MoO_3__(00k) one. In order to compare the electrical properties of the electrodes, an electric equivalent circuit was proposed, as shown in the inset of Fig. 5a. The simple model consist of 4 elements: R1—electrolyte resistance, R2—charge transfer resistance on the electrode/electrolyte interface, CPE1—capacitive properties of the electrode material, and W1—Warburg element (diffusional processes). The goodness of fitting of about 3–5·10^−5^ was achieved and the results are shown in Fig. 5c. The values of R1 are comparable due to the electrochemical setup, which ensures constant distances between the electrodes. However, a significant difference was observed for the R2 values, with 261 and 3 Ωcm^2^ for the MoO_3__(00k) and the MoO_3__(011)&(102) electrode, respectively. Assuming that this value is related to cation insertion from the electrolyte to the electrode material, results indicate that exposing the (001) and (102) planes to the electrolyte facilitates the intercalation phenomenon. The values of the constant phase element parameters are related to the capacitive properties of the electrode materials. The “n” values close to 0.7 suggest that it is not “pure” double-layer capacitance, but rather a more complex modeled process^35^, thus the electric equivalent circuit used is simplified. Nevertheless, the higher “P” value indicated a higher capacitance of the MoO_3__(011)&(102) material. The values of the Warburg coefficients obtained from modeling are not exactly the same as those from the analysis shown in Fig. 5b, however, the trend remains consistent. The apparent diffusion coefficient of Al^3+^ ions was estimated using a formula derived by rearranging the definition of the Warburg coefficient, assuming that the diffusion coefficients of intercalation and deintercalation are the same:

1 $${D}_{{Al}^{3+}}={\left(\frac{RT}{\sqrt{2}{n}^{2}\sigma c{F}^{2}}\right)}^{2}$$

where R—gas constant [J·K^-1^·mol^-1^], T—temperature [K], n—charge of ion, σ—Warburg coefficient [Ωcm^2^s^−0.5^], c—concentration of ions [mol·cm^−3^] (it is assumed that c = 1 M), F—Faraday constant [C·mol^-1^].

**Figure 6:**
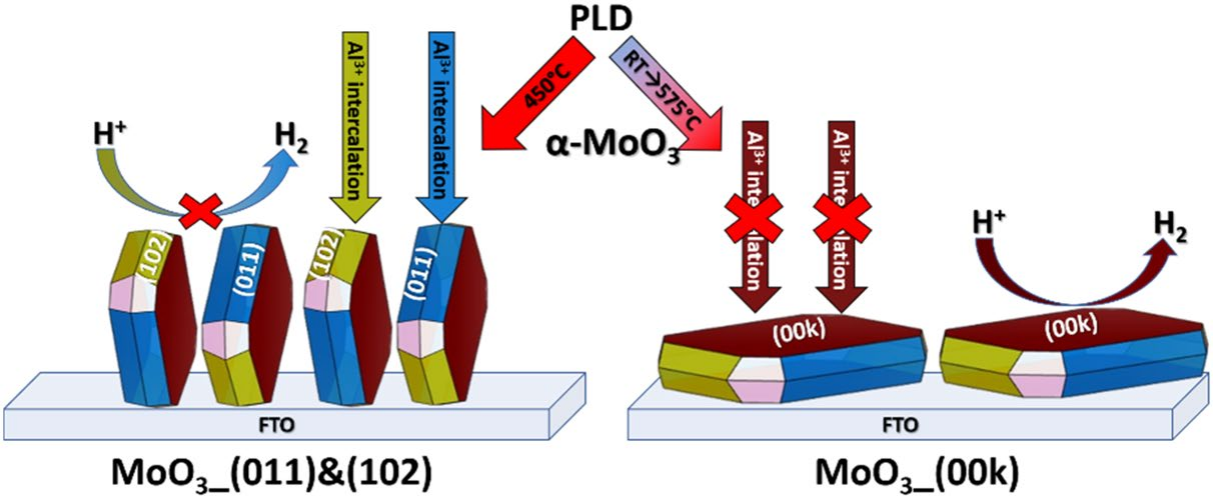
Schematic representation of the electrochemical properties of MoO_3_ films based on the orientation of the crystallites and deposition parameters.

The apparent diffusion coefficient of Al^3+^ ions estimated using the Warburg coefficient from modeling was found to be 1.9·10^−14^ and 3.9·10^−15^ cm^2^s^−1^ for the MoO_3__(011)&(102) and MoO_3__(00k) electrodes, respectively. Notably, different diffusion coefficients were determined for electrodeposited MoO_3_, depending on the size and charge of the cation; for instance, D_Na+_ was higher than D_Al_^3+^^36^. Moreover, the values of D_Al_^3+^ are in good agreement with values presented here. In this work, the diffusion coefficients of the same cation were compared for polycrystalline films with differently oriented crystallites. Moreover, it can be expected that intercalated ions are mobile in vdW gaps. According to the results, in the case of the polycrystalline film with vdW gaps perpendicular to the substrate, diffusion is facilitated, resulting in a higher diffusion coefficient compared to the material with vdW gaps parallel to the substrate. Furthermore, the values of charge transfer resistance suggest that the intercalation phenomenon occurs on the (011) and (102) facets, while the mobility of the cation through the (00k) plane is hindered. On the other hand, the electrode material with exposed (00k) planes exhibits enhanced electrocatalytic properties towards the hydrogen evolution reaction. The presented conclusions are schematically shown in Fig. 6.

## Summary

The pulsed laser deposition technique enables the deposition of MoO_3_ polycrystalline films onto FTO substrates. By adjusting the procedure parameters, the orientation of the crystallites in relation to the substrate can be controlled, without altering the substrate itself. In order to expose the (011) and (102) facets, the deposition process was carried out at 450 °C, while filling the deposition chamber with argon during the initial minutes of sputtering. Thus, the pre-deposited thin layer of metallic molybdenum promote the growth of MoO_3_ crystallites in a manner that exposes the desired facets. On the other hand, the deposition of amorphous layers followed by rapid heating at 575 °C for a short duration resulted in layers with exposed (00k) planes. In conclusion, the deposition of α-MoO_3_ on commercially available polycrystalline FTO substrates is achievable, and the orientation of the crystallites, as well as the exposure of specific crystal facets can be controlled by adjusting the deposition and post-treatment conditions. Electrochemical findings indicated that the intercalation of cations is facilitated when the vdW gaps are perpendicular to the substrate and the (011) and (102) facets are exposed to the electrolyte. Consequently, such electrode material possesses desirable properties suitable for utilization in energy storage systems, including ion batteries and electrochemical capacitors. However, when (00k) planes are exposed, electrode material exhibited improved electrocatalytic properties for hydrogen evolution, which is an equally crucial application nowadays. The apparent diffusion coefficient value also strictly depends on the deposition conditions, with the highest value of 1.9·10^−14^ cm^2 ^s^−1^ observed for the MoO_3__(001)&(102) material. Furthermore, these results show promise and warrant further exploration of the proposed procedures to assess their universality and applicability to different materials and substrates. Such investigation would provide significant benefits by enabling the study of the influence of crystallite orientation on layer properties, independent of substrate variations. This comprehensive approach would facilitate a deeper understanding of the fundamental relationships between crystallographic orientation and material functionality, fostering advancements in materials science and providing opportunity for innovative applications across various fields.

### Supplementary Information

Supplementary Information.

## Data Availability

XRD patterns and polarization curves are available in the BRIDGE OF KNOWLEDGE repository: https://mostwiedzy.pl/pl/open-research-data/xrd-and-electrochemical-results-for-moo3-films-deposited-using-pulsed-laser-deposition-system,703093455755919-0.
