# Supplementary material for: Controlling crystallites orientation and facet exposure for enhanced electrochemical properties of polycrystalline MoO3 films
Source: Sci Rep. 2023 Oct 4;13:16668. doi: 10.1038/s41598-023-43800-9 (PMC10550991; doi:10.1038/s41598-023-43800-9)
Supplement: Supplementary file 1 — Supplementary Information. [file 41598_2023_43800_MOESM1_ESM.docx]

**Controlling Crystallites Orientation and Facet Exposure for Enhanced Electrochemical Properties of Polycrystalline MoO_3_ Films**

Konrad Trzciński^a,b^*, Zuzanna Zarach^a^, Mariusz Szkoda^a,b^, Andrzej P. Nowak^a^, Katarzyna Berent^d^, Mirosław Sawczak^c^

^a^Faculty of Chemistry, Gdańsk University of Technology, Narutowicza 11/12, 80-233 Gdańsk, Poland

^b^Advanced Materials Center, Gdańsk University of Technology, Narutowicza 11/12, 80-233 Gdańsk, Poland

^c^Centre for Plasma and Laser Engineering, The Szewalski Institute of Fluid Flow Machinery, Fiszera 14, 80-231, Gdańsk, Poland

^d^AGH University of Krakow, Academic Centre for Materials and Nanotechnology, Mickiewicza 30 Ave., 30-059, Kraków, Poland

*corresponding author: kontrzci@pg.edu.pl


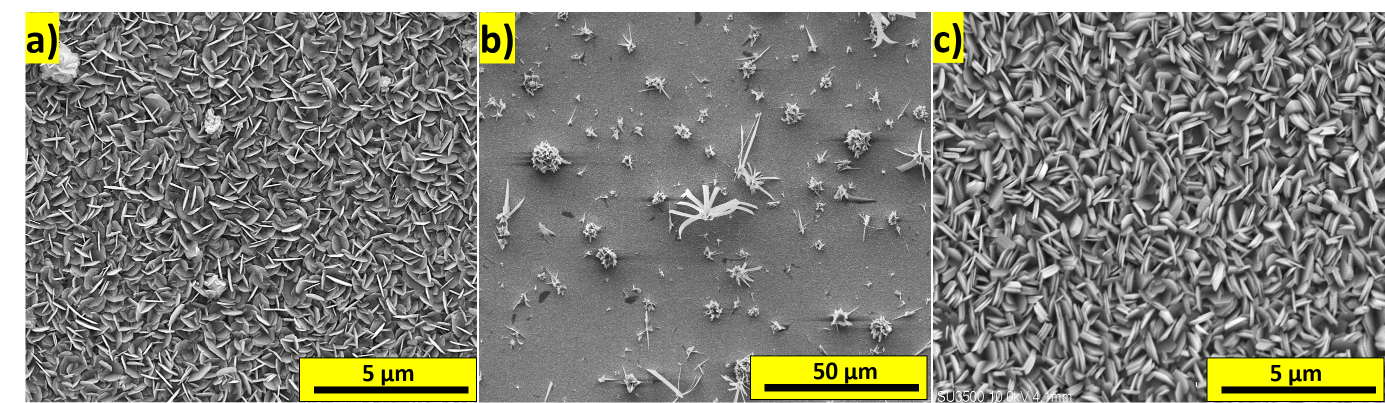


**Fig. S1** SEM micrographs of MoO_3_ deposited on the FTO substrate at a) 400°C and b) 500°C under 0.5 mbar O_2_ for 120 minutes; c) SEM micrograph of MoO_3_ deposited on FTO/Mo substrate (Mo deposited on FTO using magnetron sputtering system), performed at 450°C under 0.5 mbar O_2_ for 120 minutes.


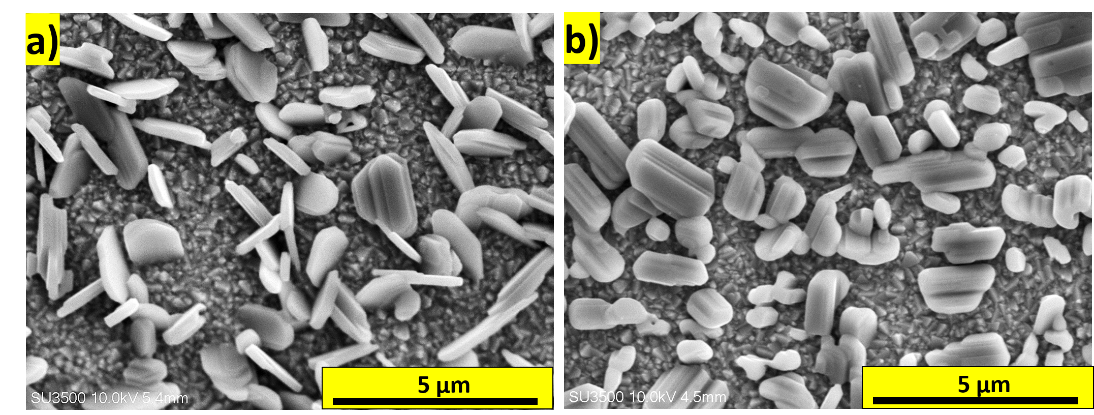


**Fig.S2** SEM micrographs of a) MoO_3_ deposited at room temperature (120 minutes) with subsequent annealing for 4 hours at 450°C and b) MoO_3_ deposited at room temperature (120 minutes) with subsequent “rapid” annealing for 20 minutes at 575°C.


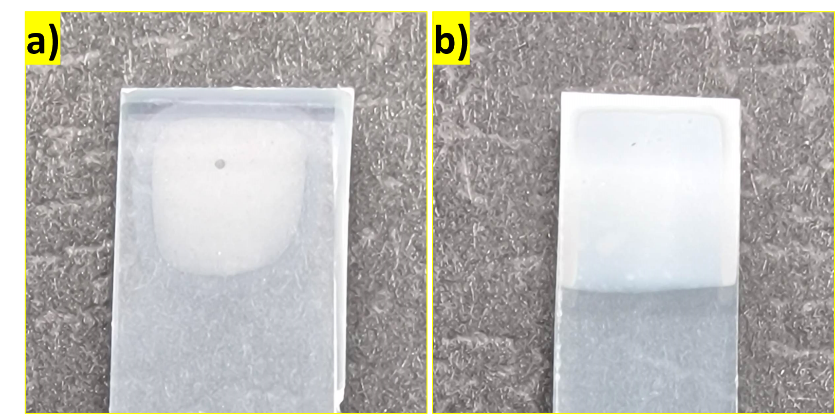


**Fig. S3** Digital photos of samples deposited at room temperature (120 minutes) with subsequent annealing for a) 60 minutes and b) 5 minutes at 575°C (MoO_3__(00k)).


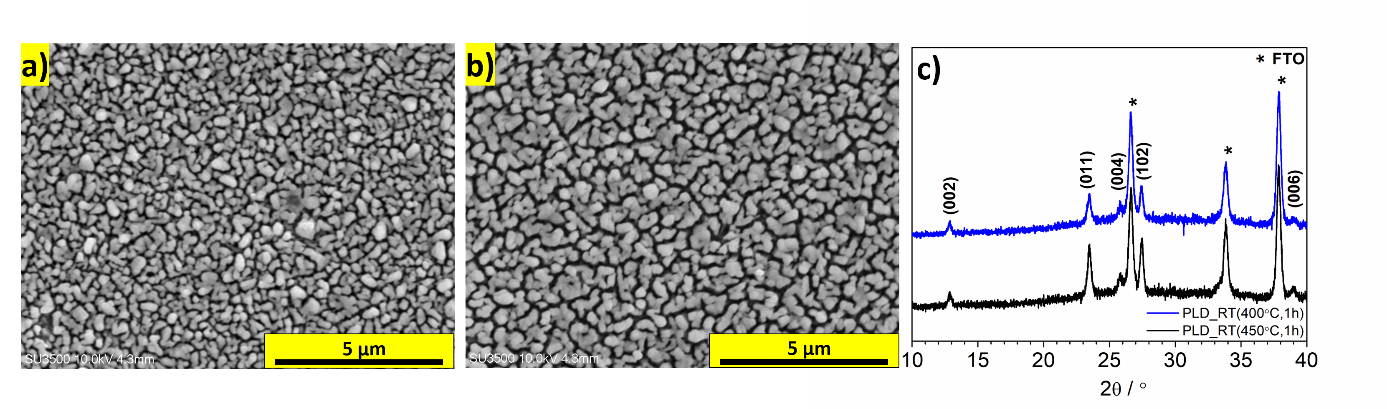


**Fig. S4** SEM micrographs of MoO_3_ films deposited at room temperature (120 minutes) with subsequent “rapid” annealing for 1 hour at a) 400°C and b) 450°C; c) XRD patterns of both samples.


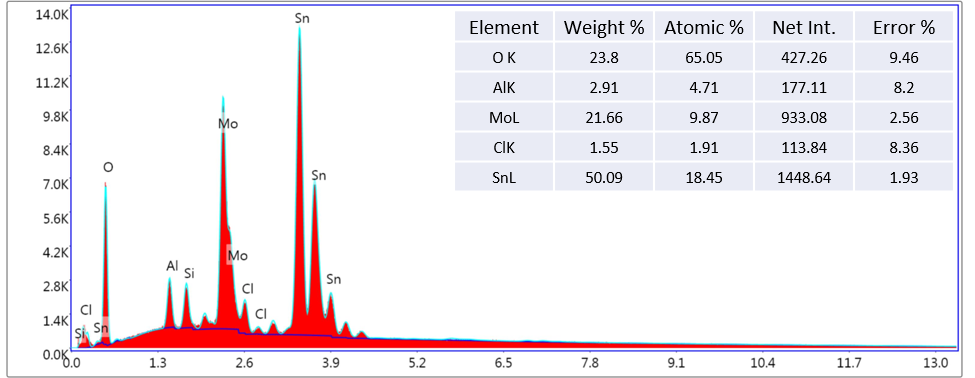


**Fig. S5** EDX results for the MoO_3__(001)&(102) electrode material after cathodic polarization at -0.1 V vs. Ag/AgCl (3 M KCl).
